# Molecular and Pathogenic Characterization of *Cylindrocarpon*-like Anamorphs Causing Root and Basal Rot of Almonds

**DOI:** 10.3390/plants11070984

**Published:** 2022-04-04

**Authors:** Nieves Capote, María Ángeles Del Río, Juan Francisco Herencia, Francisco Teodoro Arroyo

**Affiliations:** Andalusian Institute of Agricultural and Fisheries Research and Training (IFAPA) Centro Las Torres, 41200 Seville, Spain; 1mdelrioverdugo@gmail.com (M.Á.D.R.); juanf.herencia@juntadeandalucia.es (J.F.H.); franciscot.arroyo@juntadeandalucia.es (F.T.A.)

**Keywords:** *Prunus dulcis*, *Dactylonectria*, *Ilyonectria*, *Neonectria*, *Cylindrocladiella*, nursery, phylogenetic analysis, pathogenicity test

## Abstract

Three almond nurseries were prospected in the South of Spain (Sevilla) to evaluate the sanitary status of the nursery plant material. Samples consisted of main roots, secondary roots and six-month-old basal stems ‘GxN-15’, ‘Nemaguard’, ‘Cadaman’, ‘Rootpac-40’ and ‘Rootpac-20’ rootstocks planted in the soil, and twigs of mother plants from ‘Lauranne’, ‘Guara’, ‘Marcona’, ‘Marta’ and ‘Ferragnes’ almond cultivars. Endophytic and potential pathogenic fungi were identified in mother plants and 70 *Cylindrocarpon*-like anamorph isolates were detected in the root system and basal stems of analyzed rootstocks. Based on partial sequencing of the *his3* gene and multilocus phylogenetic analysis of the concatenated ITS, *tub2*, *his3* and *tef1-α* partial sequences, seven *Cylindrocarpon*-like anamorph species were identified as *Dactylonectria torresensis*, *D. novozelandica*, *D. macrodidyma*, *Ilyonectria liriodendri, Neonectria* sp. 1, *N. quercicola* and *Cylindrocladiella variabilis.* Pathogenicity was assessed on young healthy detached twigs of ‘Guara’ almond cultivar and one-year-old ‘Lauranne’ potted almonds grafted onto ‘GxN-15’ rootstocks. Among the seven *Cylindrocarpon*-like anamorph species, *I. liriodendri, Neonectria* sp. 1 and *N. quercicola* were the most aggressive. Inoculated detached shoots developed necrotic lesions 15 days after inoculation. Inoculated trees showed sectorized necrosis in the main and secondary roots and the basal stem of the rootstock 5 months after inoculation. The most aggressive species were able to cause necrosis also in the grafted cultivar, and *I. liriodendri,* and *N. quercicola* also reduced the root biomass. This is the first report of *Cylindrocarpon*-like anamorph species causing root and basal rot of almonds.

## 1. Introduction

Almond, *Prunus dulcis* (Mill.) D.A. Webb, is one of the most important nut crops, cultivated mainly in Mediterranean countries, California (USA), South Africa and Australasia. Spain is the second largest producer of almonds after California, displaying 718,540 ha of cultivation and a production of 421,610 t [1]. Andalusia, Southern Spain, contains about 28% of the almond cultivation of Spain, 200 thousand ha in 2019, most of them under rainfed conditions and with a production of around 110 thousand tons [1]. Almond cultivation and consumption have undergone a great boost in recent years, due to the profitability of the crop and the nutritional, functional and healthy properties of almond seeds [2]. In Spain, the traditional rainfed crop is being transformed into a highly technical crop, with the implementation of fertirrigation, mechanized management and the use of new varieties [3,4]. This new scenario has caused a significant increase in the production yields, but also the emergence and re-emergence of new fungal diseases [5,6]. Some of them are transmitted through asymptomatic nursery plant material [7].

*Cylindrocarpon*-like anamorphs are worldwide distributed soil-borne fungi considered saprophytic or weak pathogens [8], although they can cause cankers, root rot and decay of many herbaceous and woody plants with high economic importance, including grapevine [9,10,11], olive [12], loquat [13], kiwifruit [14], apple [15,16], peach [17,18], avocado [19], strawberry and raspberry [20], and forest trees [21,22]. *Cylindrocarpon*-like anamorphs can infect plant roots and stems via wounds or natural openings. Soil is an important source of inoculum for *Cylindrocarpon* pathogens [23] since most species can produce conidia able to spread in soil water and resistance structures, named chlamydospores, that allow them to survive in the soil for long periods [24]. In addition, infected nursery plant material is another important source of inoculum, as these species could be isolated from rootstock mother plants, rooted rootstocks, grafting material and young plants from nurseries [20,22,25,26,27,28,29].

The genus *Cylindrocarpon* (*Sordariomycetes, Hypocreales, Nectriaceae*) was first described by Wollenweber in 1913 as the asexual form of the sexual genus *Neonectria* [30]. Its taxonomy, based on morphological and phylogenetic analyses, has suffered numerous changes over time [8,31,32,33,34,35,36,37,38,39]. Currently, *Cylindrocarpon*-like anamorphs are considered a group that includes species of the genera *Campylocarpon*, *Cylindrocladiella*, *Cylindrodendrum*, *Dactylonectria*, *Ilyonectria*, *Neonectria*, *Pleiocarpon* and *Thelonectria.* Although some of these species have been associated with woody crops diseases, there is a lack of information on the pathogenicity of *Cylindrocarpon*-like anamorphs on almonds. To date, *Neonectria ramulariae* (anamorph: *Cylindrocarpon obtusiusculum*) has been associated with canker disease in cold-storage fruit and nut tree seedlings in California [40], and *D. macrodidyma* has been associated with almond trees in California with no pathogenic assessment [38,41]. In other nut crops, *Cylindrocarpon destructans* (renamed as *Ilyonectria destructans*) has been reported as a pathogen in English walnut in Italy [42] and *D. macrodidyma*, *D. novozelandica*, *D. torresensis*, *N. californica* and *Thelonectria aurea* has been associated to root rot symptoms in pistachio trees [38] but their pathogenicity has not been confirmed. Thus, the objectives of this study were (i) to conduct surveys in almond nurseries to evaluate the sanitary status of the nursery plant material, and (ii) to molecularly identify and pathogenically characterize the *Cylindrocarpon*-like anamorphs associated with root and basal rot of almond nursery rootstocks.

## 2. Results

### 2.1. Fungal Isolation

Endophytic and potentially pathogenic fungi were identified in nursery mother plants of the cultivars ‘Lauranne’, ‘Guara’, ‘Marcona’, ‘Marta’ and ‘Ferragnes’, mainly belonging to genera *Alternaria*, *Arthrinium*, *Aureobasidium*, *Chaetomium*, *Epicoccum*, *Nigrospora*, *Peniophora*, *Phoma* and *Spegazzinia*. In the analyzed rootstocks, a total of 70 *Cylindrocarpon*-like anamorph isolates were obtained. The incidence of *Cylindrocarpon*-like anamorphs in the three analyzed nurseries was 0.1%, 4% and 10%, respectively. ‘GxN-15’ rootstock presented the highest percentage of isolates of *Cylindrocarpon*-like species (60%), followed by ‘Rootpac-40’ (21%), ‘Rootpac-R’ (10%), and ‘Nemaguard’ (6%) rootstocks. No *Cylindrocarpon*-like anamorphs were detected in ‘Cadaman’ rootstock. *Cylindrocarpon*-like species were more frequently isolated from the main root (61%), and then from the secondary roots (36%) and the basal trunk (3%) (Table 1). 

### 2.2. Species Identification 

**Figure 1:**
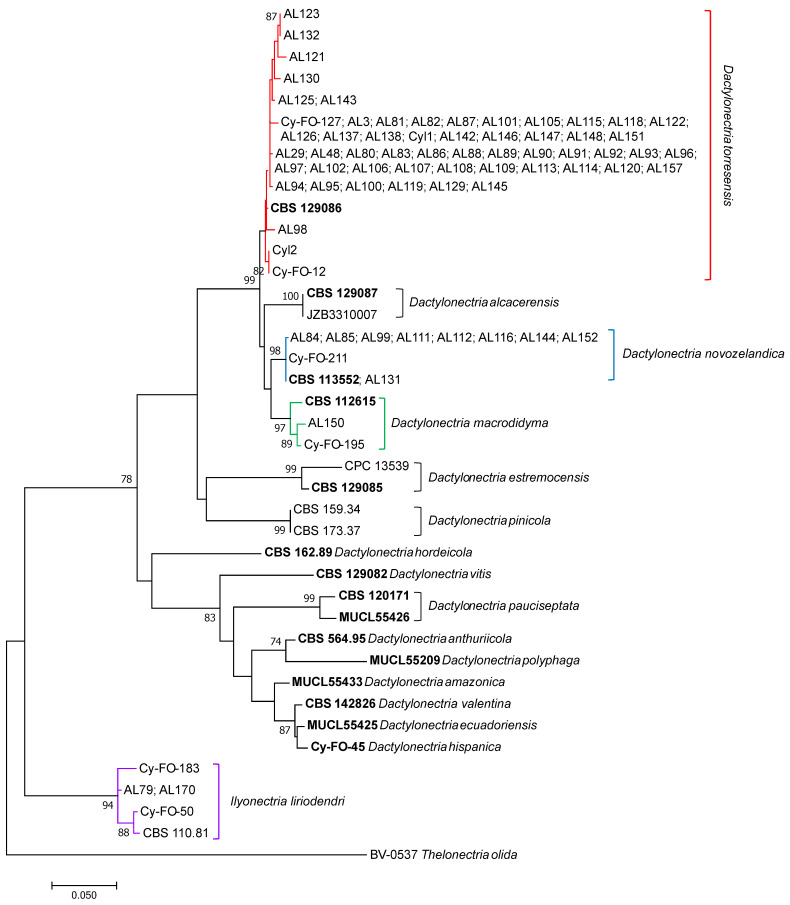
Phylogenetic Maximum Likelihood tree of *Cylindrocarpon*-like anamorphs isolated from nursery almond rootstocks and retrieved from the Genbank (ex-type cultures in bold), based on the *his3* partial sequences (617 positions including gaps) using the general-time-reversible (GTR) model with a rate of variation across sites (+G) and a proportion of invariable sites (+I). Support for internal branches was assessed by 1000 mL bootstrapped pseudoreplicates of data. Nodes with bootstrap support higher than 70% were indicated in the final tree. The tree with the highest log likelihood is shown. The tree was rooted to *Thelonectria olida* BV-0537 isolate. The scale bar indicates 0.05 expected changes per site. Evolutionary analyses were conducted in MEGA7.

**Figure 2:**
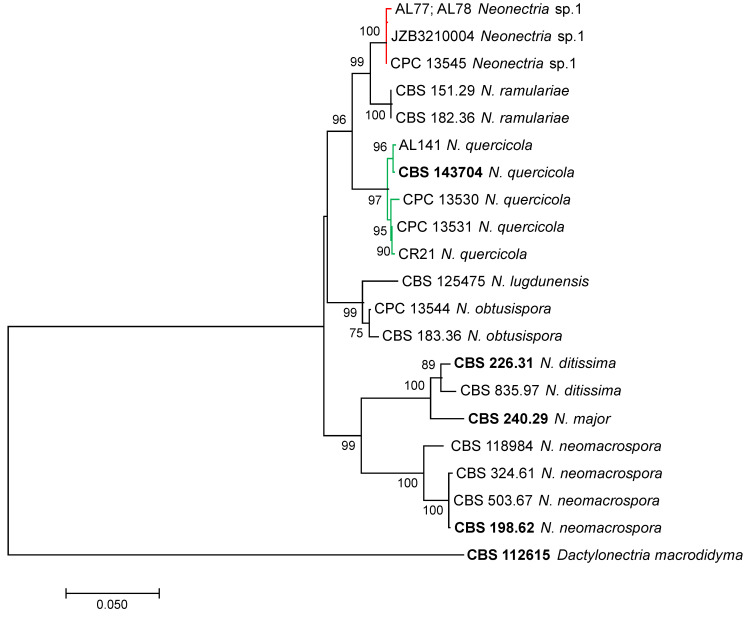
Phylogenetic Maximum Likelihood tree of *Neonectria* spp. isolated from nursery almond rootstocks and retrieved from the Genbank (ex-type cultures in bold), based on the concatenated partial sequences of ITS, *tub2*, *his3* and *tef1-α* (2341 positions) using the Tamura-3 parameter model with a rate of variation across sites (+G). Support for internal branches was assessed by 1000 mL bootstrapped pseudoreplicates of data. Nodes with bootstrap support higher than 70% were indicated in the final tree. The tree with the highest log likelihood is shown. The tree was rooted to *Dactylonectria macrodidyma* (CBS 112601). The scale bar indicates 0.05 expected changes per site. Evolutionary analyses were conducted in MEGA7.

*Dactylonectria torresensis*, *D. macrodidyma*, *D. novozelandica*, *Ilyonectria liriodendri* and *Cylindrocladiella variabilis* species were identified based on the partial sequencing and phylogenetic analysis of the *his3* gene. The *his3* tree allowed the separation of *D. torresensis*, *D. macrodidyma*, *D. novozelandica* and *I. liriodendri* species with high bootstrap values (Figure 1). An independent phylogenetic analysis of *his3* partial sequence identified AL139 isolate as *Cylindrocladiella variabilis* with 98% similarity to *Cyl. variabilis* CBS 375.93 isolate (Acc. No. JN09888) and a bootstrap value of 78% (Figure S1). *Neonectria* sp. 1 and *N. quercicola* species were identified by a multilocus phylogenetic analysis using the concatenated partial sequences of ITS, tub2, his3 and tef1-α. In the ML multilocus tree, two isolates (AL77 and AL78) clustered with Neonectria sp. 1 with bootstrap values of 100%, and the isolate AL141 clustered with *N. quercicola* with a bootstrap value of 96% (Figure 2). *Dactylonectria torresensis* was the most frequent Cylindrocarpon-like anamorph species detected in the rootstocks of nursery almonds (76.5%), followed by *D. novozelandica* (14%), *I. liriodendri* and *Neonectria* sp. 1 (3%) and *D. macrodidyma*, *N. quercicola* and *Cylindrocladiella variabilis* (1.5%).

### 2.3. Pathogenicity Tests in Detached Cultivar Twigs 

**Figure 3:**
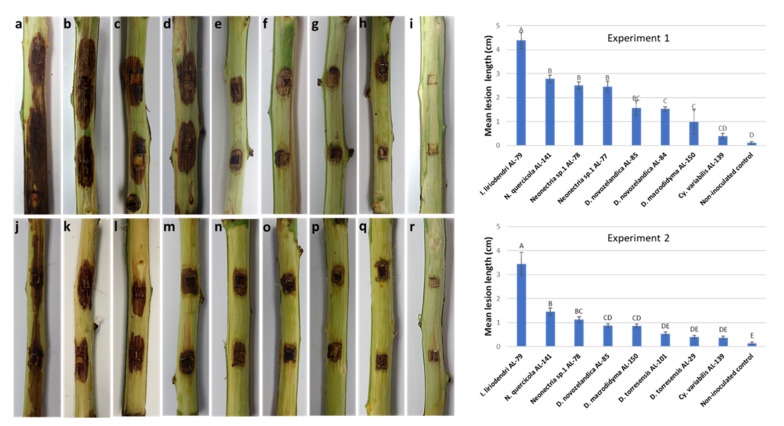
Lesions caused in detached ‘Guara’ almond twigs 15 days after inoculation with *Cylindrocarpon*-like anamorphs: *Ilyonectria liriodendri* AL79 isolate (**a**,**j**); *Neonectria quercicola* AL141 isolate (**b**,**k**); *Neonectria* sp. 1 AL78 (**c**,**l**) and AL77 (**d**) isolates; *Dactylonectria novozelandica* AL85 (**e**,**m**) and AL84 (**f**) isolates; *D. macrodidyma* AL150 isolate (**g**,**n**); *D. torresensis* AL101 (**o**) and AL29 (**p**) isolates; *Cylindrocladiella variabilis* AL139 isolate (**h**,**q**), and non-inoculated control (**i**,**r**). Two independent experiments are shown with some differences in the isolates tested. Results are the mean of 16 inoculation points (eight twigs and two wounds per twig) per isolate and error bars correspond to the standard error. Data were subjected to analysis of variance (ANOVA) and different letters indicate significant differences according to the LSD test at *p* < 0.05.

In the pathogenicity test performed on detached green twigs of ‘Guara’ almond cultivar, the non-inoculated control twigs did not develop any symptoms (Figure 3). In contrast, shoots inoculated with *Ilyonectria*, *Neonectria*, *Dactylonectria*, and *Cylindrocladiella* species resulted in the development of brown necrotic lesions at 15 days after inoculation (dai) in both experiments. The mean lesion lengths significantly differed among the inoculated species (*p* < 0.05) (Figure 3). *Ilyonectria liriodendri*, *N. quercicola* and *Neonectria* sp. 1 were the most aggressive species in both experiments causing lesions between 3.5 to 4.4 cm, 1.5 to 2.8 cm, and 1.1 to 2.5 cm, respectively. *Dactylonectria novozelandica* and *D. macrodidyma* isolates caused medium-length lesions that varied between 0.7 to 1.1 cm and 0.2 and 0.9 cm, respectively. Finally, *D. torresensis* and *C. variabilis* were the species that produced the lowest necrotic lesions in the inoculated twigs, not significantly deferring from the non-inoculated control. The re-isolation rates from the lesions varied between 90% and 100%.

### 2.4. Pathogenicity Test on Young Almond Trees 

**Figure 4:**
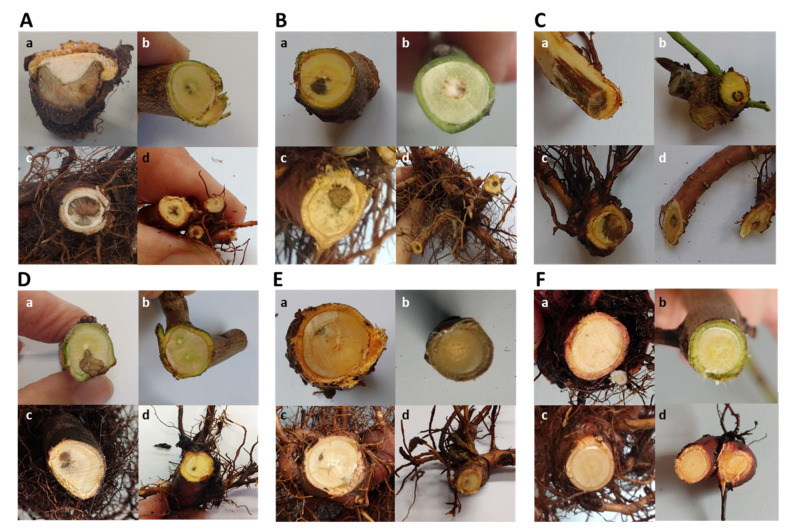
Representative internal symptoms in ‘Lauranne’ almonds grafted onto ‘GxN-15’ inoculated with (**A**) *Ilyonectria liriodendri* AL79 isolate; (**B**) *Neonectria quercicola* AL141 isolate; (**C**) *Neonectria* sp. 1 AL77 isolate; (**D***) Dactylonectria novozelandica* AL84 isolate; (**E**) *D. torresensis* AL29 isolate; (**F**) non-inoculated control plants. Symptoms in inoculated plants consisted of sectorized necrosis in basal stem (**a**); slight or no symptoms in the grafted variety (**b**); central or sectorized necrosis in the main root (**c**) and secondary roots (**d**).

**Figure 5:**
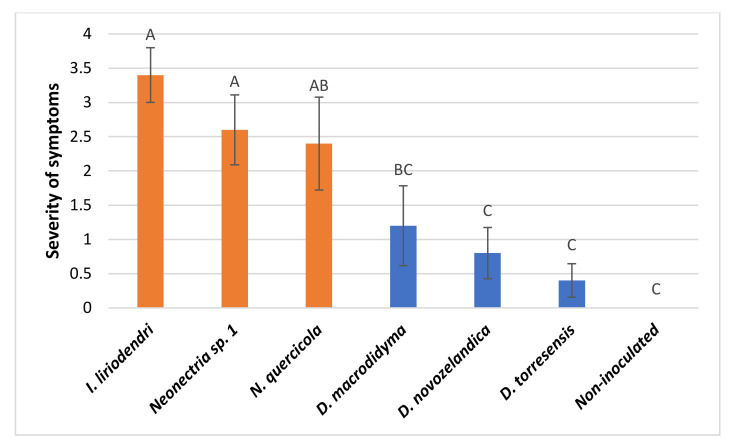
Severity of internal symptoms in ‘Lauranne’ almonds grafted onto ‘GxN-15’ inoculated with *Ilyonectria liriodendri* AL79 isolate; *Neonectria quercicola* AL141 isolate; *Neonectria* sp. 1 AL77 isolate; *Dactylonectria macrodidyma* AL150 isolate; *D. novozelandica* AL84 isolate; *D. torresensis* AL29 isolate. Non-inoculated plants were used as controls. Potted plants were maintained in greenhouse conditions and evaluations were performed 5 months after inoculation. Results are the mean of ten plants per isolate and error bars correspond to the standard error. Data were subjected to analysis of variance (ANOVA) and different letters above bars indicate significant differences according to LSD test at *p* < 0.05.

**Figure 6:**
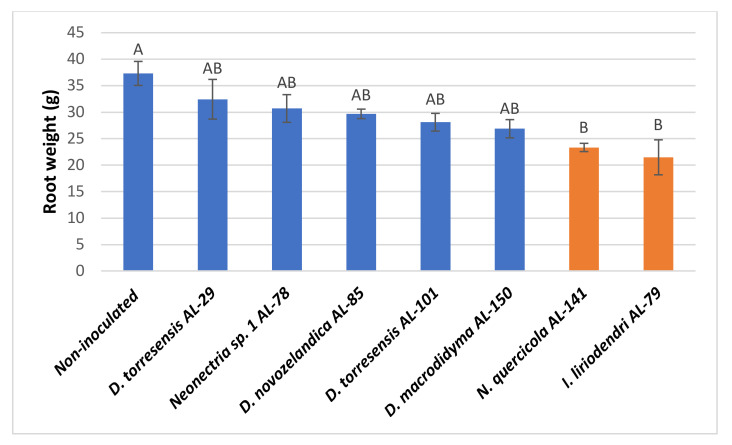
Weight of the root system of ‘Lauranne’ potted almond trees grafted onto ‘GxN-15’ rootstock inoculated with isolates of *Cylindrocarpon*-like anamorphs. Results are mean of ten trees per isolate and error bars correspond to the standard error. Data were subjected to analysis of variance (ANOVA) and different letters indicate significant differences according to LSD test at *p* < 0.05.

In the pathogenicity test performed on one-year-old ‘Lauranne’ potted almonds, all *Cylindrocarpon*-like anamorph species inoculated were able to cause necrotic lesions in the main and secondary roots and in the basal stem of the rootstocks, consisting of necrosis in sectorized areas (Figure 4A–E). The non-inoculated control trees did not develop any symptoms in the root system nor in the aerial part of the tree (Figure 4F). *Ilyonectria liriodendri* AL79, *N. quercicola* AL141, and *Neonectria* sp. 1 AL77 were the most aggressive isolates and were able to produce necrotic lesions also in the grafted cultivar (section b in Figure 4A, Figure 4B and Figure 4C, respectively). The severity of symptoms caused by *I. liriodendri* AL79, *N. quercicola* AL141, and *Neonectria* sp. 1 AL77 significantly differed from the non-inoculated plants (Figure 5). In addition, *I. liriodendri* and *N. quercicola* isolates caused a significant reduction in the root biomass of inoculated plants compared with the non-inoculated controls (Figure 6). The inoculated isolates were reisolated mainly from the main root (between 80 and 100% of the inoculated plants), then from the basal stem (0–40%) and then from the secondary roots (0–20%).

## 3. Discussion

This study demonstrates the presence of *Cylindrocarpon*-like anamorphs in plant material of almond nurseries able to cause root and basal rot. Seven *Cylindrocarpon*-like anamorph species were identified from the main and secondary roots and the basal stems of nursery rootstocks planted in the soil. The *his3* marker has been demonstrated to be essential either singly or in conjunction with ITS, *tub2* and *tef1-α* loci for the accurate identification of most *Cylindrocarpon*-like anamorphs [22,26,27], and allowed the identification of *Dactylonectria torresensis*, *D. novozelandica*, *D. macrodidyma*, *Ilyonectria liriodendri*, *Neonectria* sp. 1, *N. quercicola* and *Cylindrocladiella variabilis* species. Although *Cylindrocarpon*-like anamorphs are widely distributed and can act as endophytes and latent pathogens [9,43] they have also shown to be the causal agents of rot diseases in woody and herbaceous crops. However, there is scarce knowledge about the relevance and pathogenicity of these groups of fungi in almond crops. Fungal trunk diseases are important in rootstocks because they can be transmitted to the commercial variety, as occurred with black-foot causing pathogens detected in grapevine roots and crowns [26,44] or those causing cankers in other fruit tree species [16,17]. In this work, we have demonstrated that some *Cylindrocarpon*-like anamorphs, such as *Ilyonectria liriodendri*, *Neonectria quercicola* and *Neonectria* sp. 1 are able not only to cause root and basal rot in the rootstock but also to colonize the grafted almond cultivar. The source of inoculum of *Cylindrocarpon*-like anamorphs in almond nurseries should be investigated by the analysis of the nursery soil and plant material for cutting. In grapevine, the presence of *Cylindrocarpon*-like anamorphs associated with black-foot disease has been demonstrated in nursery soil [23] and at different stages of the grapevine nursery propagation system [29], even in asymptomatic nursery plants [26]. Therefore, the phytosanitary status of nursery plant material, the soil in which plantlets are grown, and even the irrigation water, should be thoroughly analyzed, in the frame of nurseries management, to avoid the entrance and subsequent spread of *Cylindrocarpon*-like anamorph pathogens into almond production fields.

*Dactylonectria torresensis* was the most abundant *Cylindrocarpon*-like species found in the prospected almond nurseries, although this species was the less aggressive, causing the smallest lesions in cultivar detached twigs and low symptoms in inoculated potted plants. This species is the most frequent *Cylindrocarpon*-like anamorphs associated with black-foot disease in grapevines in Italy [11], Portugal [45] and Spain [46], although *D. novozelandica* and *D. macrodidyma* proved to be the most aggressive in Spanish and Chinese grapevine nurseries, respectively, despite being detected with low frequencies [26,28]. In the same way, the only isolate detected of *Cylindrocladiella variabilis* caused minor lesions in the cultivar and was not tested in potted plants, despite some *Cylindrocladiella* species, such as *Cyl. parva* and *Cyl. peruviana*, having been associated to black-foot disease of grapevine [23].

However, we must not minimize the importance of the presence of less abundant species in nursery rootstocks, such as *I. liriodendri*, *N. quercicola* and *Neonectria* sp. 1 which are able not only capable of causing root and basal trunk rot, but also of reaching the variety and producing internal symptoms. Although symptoms observed in the grafted variety were slight, the pathogenicity tests in detached twigs confirmed that these species were able to cause extensive lesions in the ‘Guara’ almond variety. The susceptibility to *Cylindrocarpon*-like species of other almond varieties should be assessed when establishing new almond orchards. Even more, when the almond crop has experienced a boost in its cultivation, which implies the use of new varieties whose agronomic behavior is unknown, including their susceptibility to pathogens.

The accurate identification and pathogenic characterization of *Cylindrocarpon*-like anamorphs in almond nurseries will assist management decisions that could prevent or minimize infections caused by these pathogens in almond nurseries and commercial orchards.

## 4. Materials and Methods

### 4.1. Nursery Surveys and Fungal Isolation

Three almond nurseries located in southwestern Spain were prospected in May 2018 and 2019. Nurseries were located in the municipalities of La Rinconada, and Villaverde del Río (Sevilla province, Spain). Rootstocks and cultivar mother plants were independently analyzed for the detection of pathogenic fungi. Analyzed rootstocks were ‘GxN-15’, ‘Nemaguard’, ‘Rootpac-R’, ‘Rootpac-40’, ‘Rootpac-20’ and ‘Cadaman’. Analyzed mother plants were from ‘Lauranne’, ‘Guara’, ‘Marcona’, ‘Marta’ and ‘Ferragnes’ cultivars. Twenty-five plants per rootstock and cultivar were collected in each survey. Four plant pieces showing discolored or necrotic symptoms were analyzed per each rootstock tissue (main roots, secondary roots, and basal shoots), and four fragments from five asymptomatic scions were analyzed per cultivar mother plant. For fungal isolations, plant tissues were washed under tap water, surface disinfected in 1.5% sodium hypochlorite solution for 1 min, rinsed twice in sterile distilled water, and left to air dry in a laminar flow cabinet. Small pieces (0.5 cm) were plated on potato dextrose agar supplemented with 0.5 g/L of streptomycin sulfate (Sigma-Aldrich, St. Louis, MO, USA) (PDAS). The plates were incubated at 25 °C in 12-h photoperiod for 7 to 15 d. Each colony was then transferred to fresh PDA plates for further isolation. Single spore cultures were obtained from all isolates and maintained in 20% (*v*/*v*) glycerol at −80 °C in the IFAPA Las Torres fungal collection (Alcalá del Río, Sevilla, Spain). 

### 4.2. Molecular Characterization of Fungal Isolates

For DNA isolation, fungal mycelium was scraped from PDA fungal cultures, and genomic DNA was extracted using the Isolate II Plant DNA Kit (Bioline, Toronto, ON, Canada) following the manufacturer’s instructions. The DNA concentration was determined on an ND-1000 NanoDrop spectrophotometer (Nano-Drop Products, Wilmington, DE, USA). Molecular identification of fungal isolates from cultivar mother plants was performed by sequencing fragments of the internal transcribed spacer 1 and 2 (ITS), including 5.8S of the nuclear ribosomal DNA using the primers ITS1 and ITS4 [47]. To molecularly identify the *Cylindrocarpon*-like anamorphs isolated from rootstocks, partial sequencing of the histone H3 (*his3*) gene was performed using primers CYLH3F/CYLH3R [48]. Isolates identified in the GenBank database as *Cylindrocarpon* spp. were additionally sequenced for the ITS region, and partial regions of the translation elongation factor 1-α gene (*tef1-α*) using primers CylEF-1 (5′-ATGGGTAAGGAVGAVAAGAC-3′; J.Z. Groenewald, unpublished) and CylEF-R2 [48], and β-tubulin (*tub2*) using primers T1 [49] and Bt2b [50]. PCR amplifications contained 10× PCR Buffer (Intron Biotechnology, Inc., Seongnam, Gyeonggi, Korea), 2 mM MgCl_2_, 0.25 mM each dNTP, 0.4 µM each primer, 1 U of i-*Pfu* high fidelity DNA polymerase (Intron Biotechnology, Inc., Seongnam, Gyeonggi, Korea) and 20–100 ng of genomic DNA in a 25 mL final volume. Amplifications were carried out at 95 °C for 3 min followed by 35 cycles of 30 s at 95 °C, 30 s at 55 °C (for ITS) or 60 °C (for *his3*) or 59 °C (for *tef1-α*) or 52 °C (for *tub2*) and 30 s at 72 °C. Amplicon sizes were resolved in 1.5% agarose gels in 0.5 × TAE (Tris-acetate-EDTA) buffer stained with RedSafe nucleic acid staining solution (Intron Biotechnology, Inc., Seongnam, Gyeonggi, Korea) and visualized over an ultraviolet transilluminator. Amplicons were purified using the FavorPrep Gel/PCR purification kit (Favorgen, Ping-Tung, Taiwan) following the manufacturer’s instructions and sequenced in both directions by STAB VIDA DNA Sequencing Service (Caparica, Portugal). The sequences generated were deposited in the GenBank (Table 1) and compared with available sequences by BLAST analysis. One or two isolates were selected from each species for pathogenicity tests.

### 4.3. Phylogenetic Analyses

The identification of the *Cylindrocarpon*-like anamorphs was performed by partial sequencing of the *his3* gene. BLASTn analysis revealed the presence of *Dactylonectria*, *Ilyonectria*, *Neonectria* and *Cylindrocladiella* spp. A single-locus phylogenetic analysis using *his3* partial sequences (617 nt) was performed for *Dactylonectria*, *Ilyonectria* and *Cylindrocladiella* isolates. In addition, a multilocus phylogenetic analysis consisting of concatenated partial sequences of ITS, *tub2*, *his3* and *tef1-α* was performed to better resolve *Neonectria* species. Multiple sequence alignments were performed in MEGA7 software [51] using the CLUSTALW algorithm [52] refined with MUSCLE [53] and edited manually. Phylogenetic analyses were conducted using MEGA7 through maximum likelihood (ML) analysis. 

### 4.4. Pathogenicity Tests in Detached Twigs of Almond Cultivar

One-year twigs from the ‘Guara’ almond cultivar were inoculated with ten representative isolates of the *Cylindrocarpon*-like anamorph species detected in nursery plants. For this, the almond twigs were cut into 12 cm pieces, surface sterilized by spraying to run off with 75% ethanol and left to air dry in a laminar flow cabinet. Two wounds per twig were made with a 5 mm diameter cork borer. Five-mm mycelium agar plugs from PDA active growing colonies were placed with the mycelium attached to the subcortical tissue and sealed with parafilm. Inoculated twigs were individually inserted in glass tubes containing 2 mL of sterile water. Tubes were capped and incubated in a growth chamber at 25 °C in the dark. Eight twigs per isolate were inoculated. Plugs of sterile PDA were used to inoculate shoots used as controls. The experiment was performed twice in 2018 with some variations in the isolates tested. Two weeks after inoculation the twigs were peeled, and the length of the internal lesions was measured. Re-isolation of the inoculated fungal isolates were performed for all inoculated twigs by taking small pieces from the margin of the lesions and placing them onto PDA. Isolations were also performed from asymptomatic twigs. Plates were incubated at 25 °C in the dark for 10 to 15 d, and fungal growths were identified based on cultural and morphological characteristics. The percentage of re-isolation was calculated. The lesion length data were statistically analyzed with Statistix v.9.0 (Analytical Software, Tallahassee, FL, USA). To determine whether the data obtained followed a normal distribution, the Shapiro–Wilk test (W test) was used. The homogeneity of the variance of the dataset was assessed using Levene’s test. One-way ANOVA analysis was performed to evaluate the significant differences in the lesion lengths caused by each fungal species, using the LSD test for the comparisons of the means, at *p* < 0.05. 

### 4.5. Pathogenicity Tests in Potted Plants

Fungal inoculums were prepared by infecting wheat seeds. For this, seeds were soaked in water for 12 h and air dried. Glass flasks were half filled with the seeds and sterilized at 121 °C for 60 min for three consecutive days. Five-mm mycelium agar plugs from colonies actively growing in PDA for 10–15 d were transferred to the flasks in sterile conditions. Negative controls consisted of wheat seeds with non-colonized PDA plugs. Flasks were incubated at 25 °C in the dark for 1 month and shaken every week to avoid the seeds becoming compacted. Inoculated seeds were mixed with sterile peat substrate at 1%. 

Plants consisted of one-year-old almonds cv. Lauranne grafted onto ‘GxN-15’ rootstocks. Five plants per isolate were transplanted to plastic pots (3L) filled with the inoculated substrate or the negative control and maintained in greenhouse conditions for 5 months. Internal wood and root symptoms were evaluated at the end of the assays using a 0–4 scale where 0 = no symptoms; 1 = 1–25% root and basal trunk necrosis, 2 = 26–50% root and basal trunk necrosis, 3 = 51–75% root and basal trunk necrosis, and 4 = >76% root and basal trunk necrosis or necrosis in the grafted variety. In addition, the dry weights of roots were recorded for each plant. Re-isolation of the inoculated fungal isolates was performed by tissue culturing of the symptomatic tissues as described previously. The experiment was performed twice, in 2019 and 2020. Analysis of variance indicated that the data between the two repetitions were similar (*p* > 0.05). Thus, data from both experiments were combined. ANOVA analysis was performed on symptoms data and root dry weights using the LSD for the comparisons of the means, at *p* < 0.05. Data were analyzed using Statistix v.9.0. 

## 5. Conclusions

This is the first report of *Cylindrocarpon*-like anamorphs causing root and basal rot of almonds. The accurate identification and pathogenic characterization of *Cylindrocarpon*-like anamorphs in almond nurseries could be essential to aid management decisions that could prevent or minimize infections caused by these pathogens in almond nurseries and production fields. The susceptibility to *Cylindrocarpon*-like anamorphs in different almond cultivars should be investigated in further experiments.

## Figures and Tables

**Table 1:** *Cylindrocarpon*-like anamorphs used in this study.

Species	Isolate	Rootstock	Tissue of Origin	GenBank Accession No.			
				ITS	*his3*	*tub2*	*tef1-α*
*Dactylonectria torresensis*	AL3	GxN-15	main root	OM514923	OM483314	-	-
	AL29	Rootpac-40	main root	OM514924	OM483315	-	-
	AL48	GxN-15	feeder root	OM514925	OM483316	-	-
	AL80	RP-R	feeder root	OM514926	OM483317	-	-
	AL81	RP-R	basal trunk	OM514927	OM483318	-	-
	AL82	GxN-15	main root	OM514928	OM483319	-	-
	AL83	GxN-15	main root	OM514929	OM483320	-	-
	AL86	GxN-15	main root	OM514930	OM483321	-	-
	AL87	GxN-15	main root	OM514931	OM483322	-	-
	AL88	GxN-15	main root	OM514932	OM483323	-	-
	AL89	GxN-15	feeder root	OM514933	OM483324	-	-
	AL90	GxN-15	feeder root	OM514934	OM483325	-	-
	AL91	GxN-15	main root	OM514935	OM483326	-	-
	AL92	GxN-15	main root	OM514936	OM483327	-	-
	AL93	GxN-15	main root	OM514937	OM483328	-	-
	AL94	GxN-15	main root	OM514938	OM483329	-	-
	AL95	GxN-15	main root	OM514939	OM483330	-	-
	AL96	GxN-15	main root	OM514940	OM483331	-	-
	AL97	GxN-15	main root	OM514941	OM483332	-	-
	AL98	GxN-15	main root	OM514942	OM483333	-	-
	AL100	GxN-15	basal trunk	OM514943	OM483334	-	-
	AL101	GxN-15	main root	OM514944	OM483335	-	-
	AL102	GxN-15	feeder root	OM514945	OM483336	-	-
	AL105	GxN-15	main root	OM514946	OM483337	-	-
	AL106	GxN-15	main root	OM514947	OM483338	-	-
	AL107	Nemaguard	main root	OM514948	OM483339	-	-
	AL108	GxN-15	main root	OM514949	OM483340	-	-
	AL109	RP-R	feeder root	-	OM483341	-	-
	AL113	Rootpac-40	feeder root	-	OM483342	-	-
	AL114	Rootpac-40	feeder root	-	OM483343	-	-
	AL115	Rootpac-40	feeder root	-	OM483344	-	-
	AL118	Rootpac-40	feeder root	-	OM483345	-	-
	AL119	Rootpac-40	feeder root	-	OM483346	-	-
	AL120	Rootpac-40	feeder root	-	OM483347	-	-
	AL121	Rootpac-40	feeder root	-	OM483348	-	-
	AL122	Rootpac-R	main root	-	OM483349	-	-
	AL123	Rootpac-R	main root	-	OM483350	-	-
	AL125	GxN-15	feeder root	-	OM483351	-	-
	AL126	GxN-15	feeder root	-	OM483352	-	-
	AL129	GxN-15	main root	-	OM483353	-	-
	AL130	GxN-15	main root	-	OM483354	-	-
	AL132	GxN-15	main root	-	OM483355	-	-
	AL137	Rootpac-40	main root	-	OM483356	-	-
	AL138	Rootpac-40	feeder root	-	OM483357	-	-
	AL142	GxN-15	feeder root	-	OM483358	-	-
	AL143	GxN-15	main root	-	OM483359	-	-
	AL145	GxN-15	main root	-	OM483360	-	-
	AL146	GxN-15	main root	-	OM483361	-	-
	AL147	GxN-15	main root	-	OM483362	-	-
	AL148	GxN-15	main root	-	OM483363	-	-
	AL151	GxN-15	feeder root	-	OM483364	-	-
	AL157	Rootpac-R	main root	-	OM483365	-	-
	Cyl1	-	main root	OM514950	OM483366	-	-
	Cyl2	-	main root	OM514951	OM483367	-	-
*D. novozelandica*	AL84	GxN-15	feeder root	-	OM483368	-	-
	AL85	GxN-15	main root	-	OM483369	-	-
	AL99	GxN-15	feeder root	-	OM483370	-	-
	AL111	Rootpac-40	feeder root	-	OM483371	-	-
	AL112	Rootpac-40	feeder root	-	OM483372	-	-
	AL116	Rootpac-40	main root	-	OM483373	-	-
	AL131	GxN-15	feeder root	-	OM483374	-	-
	AL144	GxN-15	feeder root	-	OM483375	-	-
	AL152	Rootpac-R	main root	-	OM483376	-	-
*D. macrodidyma*	AL150	GxN-15	main root	-	OM483377	-	-
*Ilyonectria liriodendri*	AL79	Nemaguard	main root	-	OM483378	-	-
	AL170	GxN-15	main root	-	OM483379	-	-
*Neonectria* sp. 1	AL77	Nemaguard	main root	OM514952	OM483380	OM483386	-
	AL78	Nemaguard	main root	OM514953	OM483381	OM483387	OM483384
*Neonectria quercicola*	AL141	Rootpac-40	main root	OM514954	OM483382	OM483388	OM483385
*Cylindrocladiella variabilis*	AL139	Rootpac-40	feeder root	-	OM483383	-	-

Sequences retrieved from the GenBank can be consulted in Supplementary Table S1.

## Data Availability

Not applicable.

## Supplementary Materials

The following supporting information can be downloaded at: https://www.mdpi.com/article/10.3390/plants11070984/s1, Figure S1: Molecular Phylogenetic analysis of *Cylindrocladiella* spp. by Maximum Likelihood method. Table S1: Isolates retrieved from the GenBank used in this study
