# Supplementary material for: Molecular and Pathogenic Characterization of Cylindrocarpon-like Anamorphs Causing Root and Basal Rot of Almonds
Source: Plants (Basel). 2022 Apr 4;11(7):984. doi: 10.3390/plants11070984 (PMC9003061; doi:10.3390/plants11070984)
Supplement: Supplementary file 1 [file plants-11-00984-s001.zip › plants-1611154-supplementary.pptx]

## Slide 1
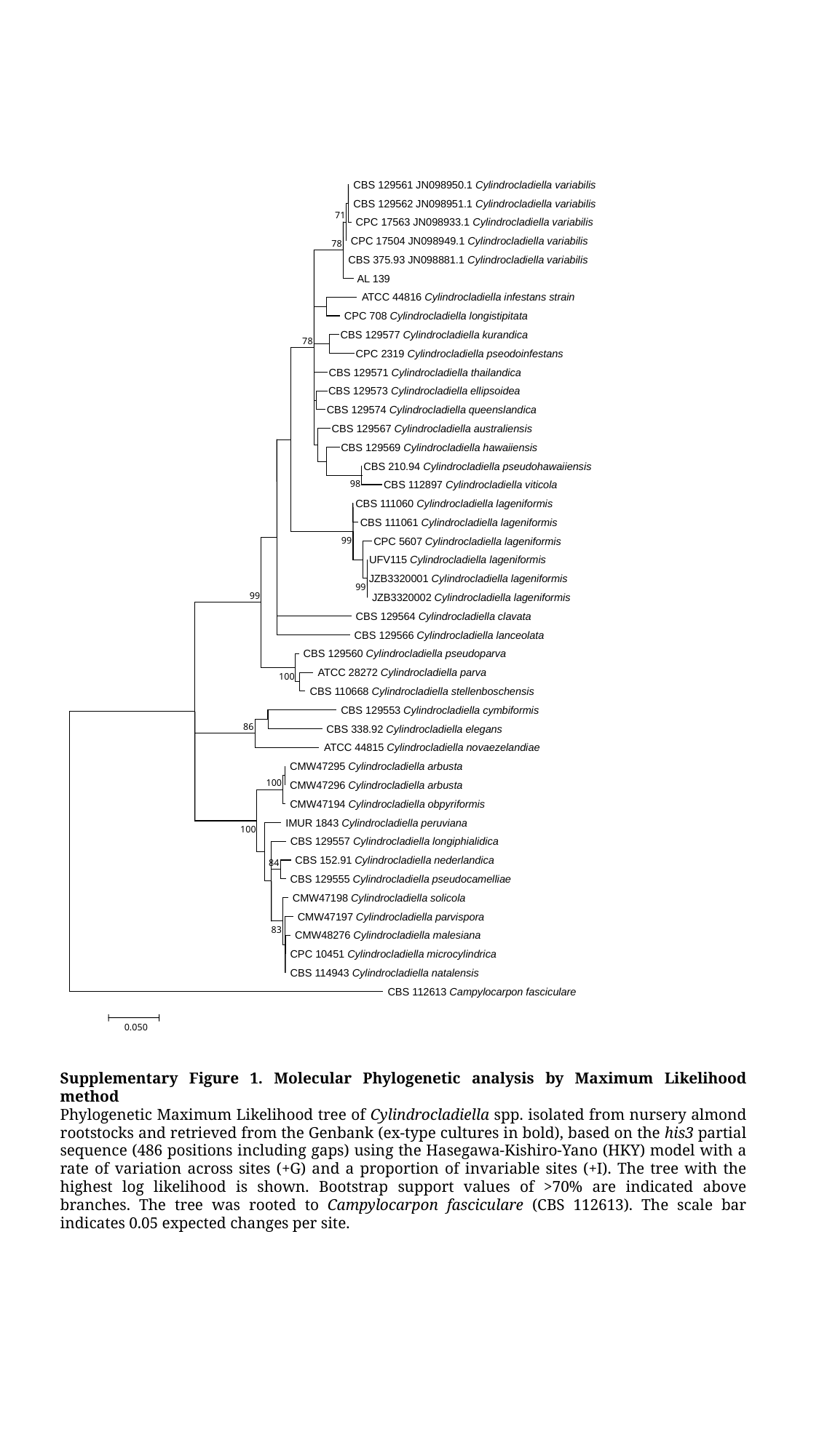

CBS 129561 JN098950.1 Cylindrocladiella variabilis
 CBS 129562 JN098951.1 Cylindrocladiella variabilis
71
 CPC 17563 JN098933.1 Cylindrocladiella variabilis
 CPC 17504 JN098949.1 Cylindrocladiella variabilis
78
 CBS 375.93 JN098881.1 Cylindrocladiella variabilis
 AL 139
 ATCC 44816 Cylindrocladiella infestans strain
 CPC 708 Cylindrocladiella longistipitata
CBS 129577 Cylindrocladiella kurandica
78
CPC 2319 Cylindrocladiella pseodoinfestans
CBS 129571 Cylindrocladiella thailandica
CBS 129573 Cylindrocladiella ellipsoidea
CBS 129574 Cylindrocladiella queenslandica
CBS 129567 Cylindrocladiella australiensis
CBS 129569 Cylindrocladiella hawaiiensis
CBS 210.94 Cylindrocladiella pseudohawaiiensis
CBS 112897 Cylindrocladiella viticola
98
CBS 111060 Cylindrocladiella lageniformis
CBS 111061 Cylindrocladiella lageniformis
CPC 5607 Cylindrocladiella lageniformis
99
UFV115 Cylindrocladiella lageniformis
JZB3320001 Cylindrocladiella lageniformis
99
99
 JZB3320002 Cylindrocladiella lageniformis
 CBS 129564 Cylindrocladiella clavata
 CBS 129566 Cylindrocladiella lanceolata
 CBS 129560 Cylindrocladiella pseudoparva
 ATCC 28272 Cylindrocladiella parva
100
 CBS 110668 Cylindrocladiella stellenboschensis
 CBS 129553 Cylindrocladiella cymbiformis
86
 CBS 338.92 Cylindrocladiella elegans
 ATCC 44815 Cylindrocladiella novaezelandiae
 CMW47295 Cylindrocladiella arbusta
100
 CMW47296 Cylindrocladiella arbusta
 CMW47194 Cylindrocladiella obpyriformis
 IMUR 1843 Cylindrocladiella peruviana
100
 CBS 129557 Cylindrocladiella longiphialidica
 CBS 152.91 Cylindrocladiella nederlandica
84
 CBS 129555 Cylindrocladiella pseudocamelliae
 CMW47198 Cylindrocladiella solicola
 CMW47197 Cylindrocladiella parvispora
83
 CMW48276 Cylindrocladiella malesiana
 CPC 10451 Cylindrocladiella microcylindrica
 CBS 114943 Cylindrocladiella natalensis
 CBS 112613 Campylocarpon fasciculare
0.050
Supplementary Figure 1. Molecular Phylogenetic analysis by Maximum Likelihood method
Phylogenetic Maximum Likelihood tree of Cylindrocladiella spp. isolated from nursery almond rootstocks and retrieved from the Genbank (ex-type cultures in bold), based on the his3 partial sequence (486 positions including gaps) using the Hasegawa-Kishiro-Yano (HKY) model with a rate of variation across sites (+G) and a proportion of invariable sites (+I). The tree with the highest log likelihood is shown. Bootstrap support values of >70% are indicated above branches. The tree was rooted to Campylocarpon fasciculare (CBS 112613). The scale bar indicates 0.05 expected changes per site.

## Slide 2
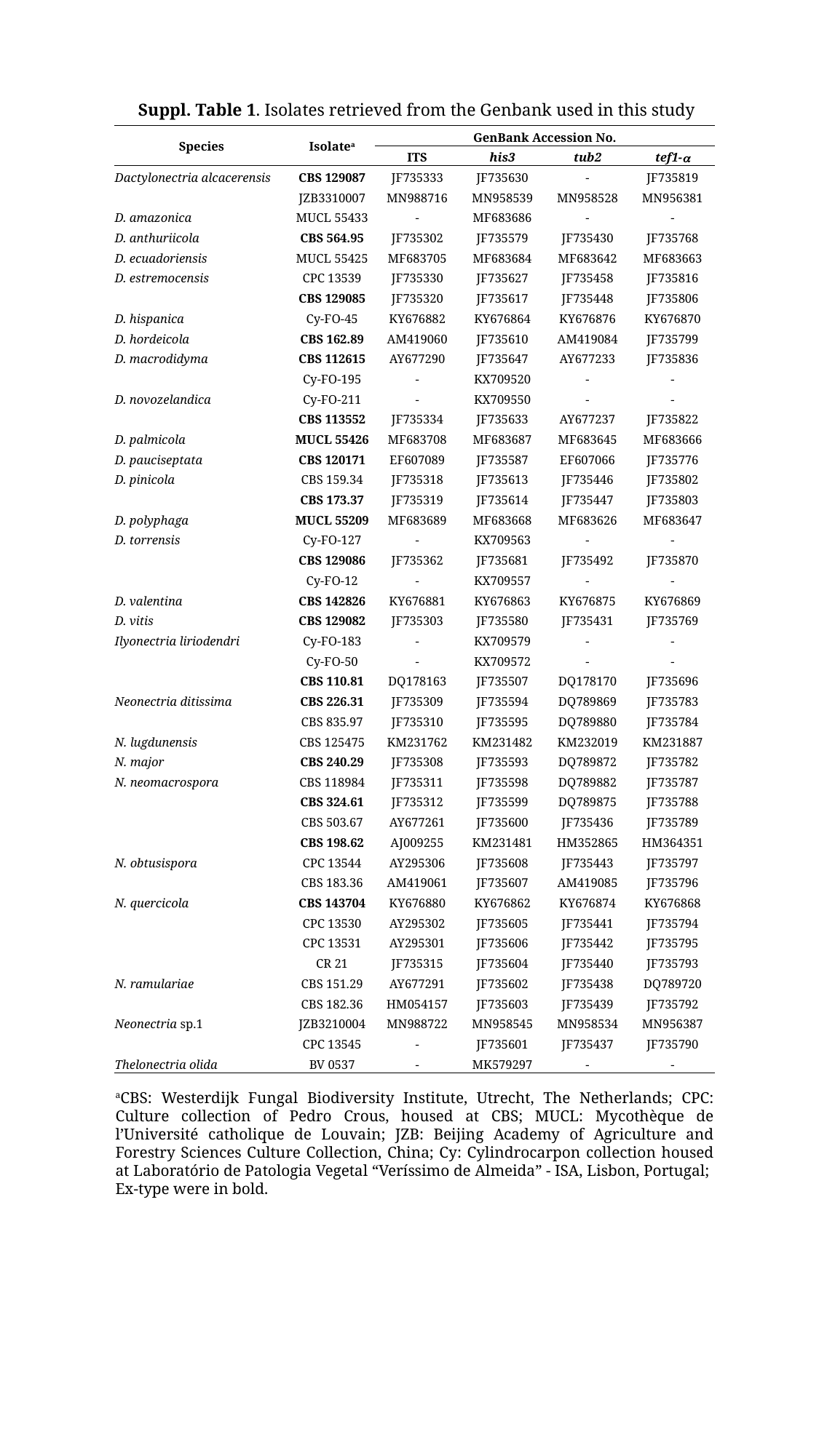

Suppl. Table 1. Isolates retrieved from the Genbank used in this study
| Species | Isolatea | GenBank Accession No. | | | |
| --- | --- | --- | --- | --- | --- |
| | | ITS | his3 | tub2 | tef1-a |
| Dactylonectria alcacerensis | CBS 129087 | JF735333 | JF735630 | - | JF735819 |
| | JZB3310007 | MN988716 | MN958539 | MN958528 | MN956381 |
| D. amazonica | MUCL 55433 | - | MF683686 | - | - |
| D. anthuriicola | CBS 564.95 | JF735302 | JF735579 | JF735430 | JF735768 |
| D. ecuadoriensis | MUCL 55425 | MF683705 | MF683684 | MF683642 | MF683663 |
| D. estremocensis | CPC 13539 | JF735330 | JF735627 | JF735458 | JF735816 |
| | CBS 129085 | JF735320 | JF735617 | JF735448 | JF735806 |
| D. hispanica | Cy-FO-45 | KY676882 | KY676864 | KY676876 | KY676870 |
| D. hordeicola | CBS 162.89 | AM419060 | JF735610 | AM419084 | JF735799 |
| D. macrodidyma | CBS 112615 | AY677290 | JF735647 | AY677233 | JF735836 |
| | Cy-FO-195 | - | KX709520 | - | - |
| D. novozelandica | Cy-FO-211 | - | KX709550 | - | - |
| | CBS 113552 | JF735334 | JF735633 | AY677237 | JF735822 |
| D. palmicola | MUCL 55426 | MF683708 | MF683687 | MF683645 | MF683666 |
| D. pauciseptata | CBS 120171 | EF607089 | JF735587 | EF607066 | JF735776 |
| D. pinicola | CBS 159.34 | JF735318 | JF735613 | JF735446 | JF735802 |
| | CBS 173.37 | JF735319 | JF735614 | JF735447 | JF735803 |
| D. polyphaga | MUCL 55209 | MF683689 | MF683668 | MF683626 | MF683647 |
| D. torrensis | Cy-FO-127 | - | KX709563 | - | - |
| | CBS 129086 | JF735362 | JF735681 | JF735492 | JF735870 |
| | Cy-FO-12 | - | KX709557 | - | - |
| D. valentina | CBS 142826 | KY676881 | KY676863 | KY676875 | KY676869 |
| D. vitis | CBS 129082 | JF735303 | JF735580 | JF735431 | JF735769 |
| Ilyonectria liriodendri | Cy-FO-183 | - | KX709579 | - | - |
| | Cy-FO-50 | - | KX709572 | - | - |
| | CBS 110.81 | DQ178163 | JF735507 | DQ178170 | JF735696 |
| Neonectria ditissima | CBS 226.31 | JF735309 | JF735594 | DQ789869 | JF735783 |
| | CBS 835.97 | JF735310 | JF735595 | DQ789880 | JF735784 |
| N. lugdunensis | CBS 125475 | KM231762 | KM231482 | KM232019 | KM231887 |
| N. major | CBS 240.29 | JF735308 | JF735593 | DQ789872 | JF735782 |
| N. neomacrospora | CBS 118984 | JF735311 | JF735598 | DQ789882 | JF735787 |
| | CBS 324.61 | JF735312 | JF735599 | DQ789875 | JF735788 |
| | CBS 503.67 | AY677261 | JF735600 | JF735436 | JF735789 |
| | CBS 198.62 | AJ009255 | KM231481 | HM352865 | HM364351 |
| N. obtusispora | CPC 13544 | AY295306 | JF735608 | JF735443 | JF735797 |
| | CBS 183.36 | AM419061 | JF735607 | AM419085 | JF735796 |
| N. quercicola | CBS 143704 | KY676880 | KY676862 | KY676874 | KY676868 |
| | CPC 13530 | AY295302 | JF735605 | JF735441 | JF735794 |
| | CPC 13531 | AY295301 | JF735606 | JF735442 | JF735795 |
| | CR 21 | JF735315 | JF735604 | JF735440 | JF735793 |
| N. ramulariae | CBS 151.29 | AY677291 | JF735602 | JF735438 | DQ789720 |
| | CBS 182.36 | HM054157 | JF735603 | JF735439 | JF735792 |
| Neonectria sp.1 | JZB3210004 | MN988722 | MN958545 | MN958534 | MN956387 |
| | CPC 13545 | - | JF735601 | JF735437 | JF735790 |
| Thelonectria olida | BV 0537 | - | MK579297 | - | - |
aCBS: Westerdijk Fungal Biodiversity Institute, Utrecht, The Netherlands; CPC: Culture collection of Pedro Crous, housed at CBS; MUCL: Mycothèque de l’Université catholique de Louvain; JZB: Beijing Academy of Agriculture and Forestry Sciences Culture Collection, China; Cy: Cylindrocarpon collection housed at Laboratório de Patologia Vegetal “Veríssimo de Almeida” - ISA, Lisbon, Portugal; Ex-type were in bold.
